# From Construction Workers to Architects: Developing Scientific Research Capacity in Low-Income Countries

**DOI:** 10.1371/journal.pbio.1000156

**Published:** 2009-07-21

**Authors:** Josefina Coloma, Eva Harris

**Affiliations:** 1 Sustainable Sciences Institute, San Francisco, California, United States of America; 2 Division of Infectious Diseases and Vaccinology, School of Public Health, University of California Berkeley, Berkeley, California, United States of America; Weatherall Institute of Molecular Medicine, United Kingdom

## Abstract

Solving global health challenges in a sustainable manner depends on explicitly addressing scientific capacity-building needs, as well as establishing long-term, meaningful partnerships with colleagues in the developing world.

Just a dozen years ago, the largest problem in tackling diseases that disproportionately affect the global South was the lack of resources available to identify and combat them. Now, as a result of the extraordinary rise in philanthropy and public giving, more funds than ever before are being directed toward pressing health issues that ravage the world's poor. However, several factors may prevent this “bubble” of generosity from realizing major improvements in global health. Not only are substantial amounts of aid being diverted from their ultimate goals by bureaucratic barriers and corruption [1], but most funds come with strings attached and must be spent according to donors' priorities, politics, and values. Many projects are planned, managed, and implemented in large part from the “North” with cooperation of local personnel and agencies. Because these projects pursue largely donor-driven agendas, they tend to reflect the donors' interests rather than those of the recipients, with two major consequences—investments in local health infrastructure and capacity building are not prioritized, and diseases and issues that are the focus of a temporary spotlight often garner the most attention and funds.

The large amounts of funds pouring into poor countries to target a few specific diseases have left programs that address traditional health indicators—such as maternal and child health and vaccination coverage—underfunded and understaffed [2], leading to a severe deterioration in overall health capacities despite increased funding. Moreover, not many global health programs include a serious investment in developing local capacity. There are relatively few examples where local infrastructure and talent are strengthened as a primary objective of the programs. Community-based organizations that train and support teachers and health care workers and offer microfinance programs to help communities meet their own needs should be highlighted (http://www.brac.net).

Research is a major driver of social and technological innovation that can lead to health and equity improvements through a knowledge-to-action process. Recognizing the need for building research capacity, health, science, and technology ministers and delegations from 60 countries attended the Global Ministerial Forum on Research for Health in Bamako, Mali in November 2008 (http://www.bamako2008.org) and drafted the Bamako Call to Action, which included a paradigm shift in global health policy. The countries agreed that at least 2% of national expenditures in health and at least 5% of development aid for the health sector would be committed to strengthening research and research capacity. The document reflects a new level of attention and a firm commitment to working with development agencies to ensure that funds are used for comprehensive health system and health research strengthening and not only for isolated projects, in keeping with the 10-year-old statement by the World Health Organization (WHO) Global Forum for Health Research that “strengthening research capacity in developing countries is one of the most effective and sustainable ways of advancing health and development in these countries and of helping correct the 10/90 Gap in health research”—referring to the fact that only 10% of health research funds are applied to the health problems of 90% of the world's population (http://www.globalforumhealth.org) [3]. How then to build research capacity in the developing world? As highlighted by an African participant at the Bamako conference, “It needs political commitment, national research strategies, budget lines, skills development, people asking nationally relevant questions, the capacity for countries to generate their own knowledge, the ability to use external knowledge, and a culture of enquiry” [4].

The best way for wealthy countries to invest in global health is to train young researchers in low-income countries and link them to the global medical, scientific, and public health communities. Although the Fogarty International Center of the United States National Institutes of Health has been a key player in helping build the capacity of researchers abroad, having funded approximately 5,000 scientists in low- and medium-income countries by supporting local investigator-led training and research programs, much more must be done, as nearly 40,000 developing-country researchers are needed to fill the current gap (http://www.fic.nih.gov/news/publications/global_health_matters/ghmnov-dec2008.pdf). Although brain drain from low- and middle-income countries occurs as many potential scientific leaders end up in the developed world, it can be reversed by harnessing the power of the scientific diaspora [5]. If each one of the approximately 1.5 million foreign-born scientists currently in the US alone were to reach out to one or two colleagues in his/her country of origin and solidify a partnership with them, real change could be achieved. With this strategy, the mother country would not only have access to individuals' embodied knowledge but also to their socio-professional networks. Several networks that provide communication, information, and coordination of functions already exist (Table 1), and joining one that matches one's interest is easier than ever. All that is necessary is interest in becoming part of a global community and a commitment to donate time. Contributing relevant scientific journal subscriptions, articles, or useful equipment and supplies; reviewing a grant or manuscript; or conducting a basic workshop can be a great way to begin.

**Table 1:**
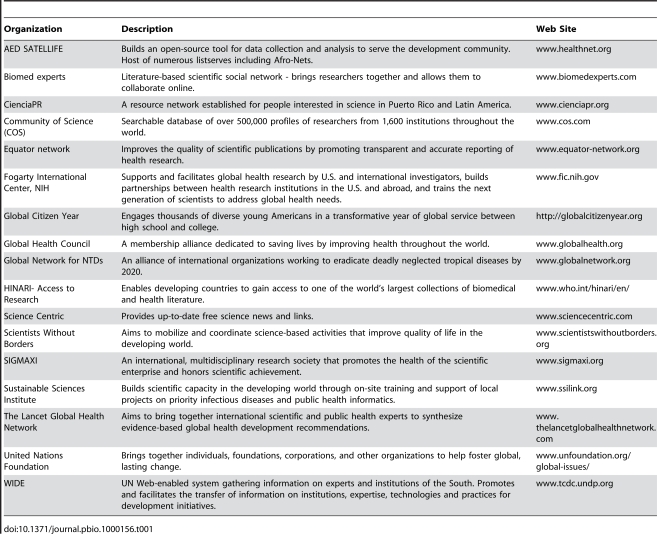
On-line networks on global health—a starting place.

The Sustainable Sciences Institute (SSI, http://www.ssilink.org), which we represent, is a non-profit organization based in San Francisco with a 20-year history of building scientific capacity in the developing world [6]. Many of its members belong to the scientific diaspora and have chosen to remain in touch with the global South as a way to give back to their countries. The working premise of the institute, which began with an emphasis on infectious diseases, is that even in low-resource settings, appropriately trained personnel with access to the knowledge and tools they need can reduce the burden of infectious diseases if they have basic resources and an essential infrastructure that supports the use of low-cost interventions.

SSI's approach to building scientific capacity is based in part on hands-on, in-country, 1- to 2-week-long workshops that fulfill specific needs expressed by colleagues in developing countries. The topics are diverse, including laboratory skills that deconstruct appropriate technologies to the basics and build them back on-site under local conditions, epidemiological methods taught within a framework that helps health professionals understand local disease patterns and design effective interventions, bioethics, bioinformatics, information and communication technologies (ICT) for health, and impact evaluation. Two additional program pillars, grant and manuscript writing, aim to increase the scientific presence and sustainability of local investigators, as well as boost their self-esteem and independence. Capacity building of human resources in a respectful and culturally appropriate manner is central to SSI's strategy in countries with very different degrees of scientific development, political contexts, and economic conditions.

To achieve long-term success, SSI provides trainees with ongoing technical, scientific, and material support through networking and consulting and material aid programs. This support allows trainees to follow their projects through to public health practice via implementation at local and national levels. All workshops are conducted in the language of the host country, and instructors include resident scientists and previous trainees from the region. Learning from researchers who are working successfully under similarly resource-constrained conditions generates trust and greater empowerment for trainees, who realize that their obstacles are not insurmountable or unique. The approach also creates a multiplier effect by generating local instructors who go on to provide further training as part of future “peer-trained” workshops, as well as ongoing training at their home institutions.

Scalability relies on the commitment of a large base of scientists and donors based in more developed countries. This model can best be illustrated by SSI's experience in Nicaragua, where long-term partnerships that began as an individual connection were followed by ongoing support that over the years translated to growth at the institutional level and, ultimately, impact on a national level. Collaboration between academic (University of California Berkeley), non-governmental (SSI), and governmental (Nicaraguan Ministry of Health) sectors has led to important advances in the field of infectious diseases and information and communication technologies for health in Nicaragua. SSI's subsidiary office in Managua now manages a number of health projects, including a multi-year pediatric dengue and influenza cohort study that is serving as a base for field and laboratory research and building the infrastructure for eventual vaccine and drug trials. To ensure adherence to the study's strict quality control procedures, SSI worked with the local study personnel and all participating institutions to raise operations to international standards in the clinic, field, and laboratory.

This project also led to the implementation of a series of low-cost information and communication technologies to streamline information flow and accessibility, improve the quality of data and quality control procedures, and reduce operational costs [7]–[9], ultimately facilitating all aspects of the study from patient flow to data management to sample and inventory tracking to laboratory procedures. These efforts, combined with hands-on training for local personnel and their colleagues, has not only facilitated study operations but has also led to development of information systems for the health sector in Nicaragua—the second poorest country in the hemisphere—using local talent and largely open-source or low-cost software that will allow for improvements in public health for years to come.

Recognizing that ICTs are important not as only technologies per se, but for the social innovation they can enable, including new ways to manage information and people to strengthen health systems, SSI is now embarking on a new initiative in health information technologies (HITs). Building on the Nicaragua experience and the current mandate to improve vaccination efficiency and prenatal care in Managua, SSI is working to identify, test, and implement low-cost, open-source ICT solutions that facilitate infectious disease research, control, and prevention in limited-resource settings. It is also evaluating the potential impacts of ICT solutions (such as electronic medical records [eHealth], mobile phone applications [mHealth], and laboratory information management systems [LIMS]) on improving targeted public health outcomes for priority health problems in underserved communities. Finally, it is strengthening partnerships and capacity-building networks in the developing world that promote knowledge exchange about sustainable best-practices in HIT implementation at a local level.

A number of other examples exist. Several European institutions have invested in capacity building in countries where their collaborators conduct field work. For example, the European and Developing Countries Clinical Trials Partnership (EDCTP, http://www.edctp.org), funded in response to the health crisis in sub-Saharan Africa, focuses on creating a critical mass of local researchers and health personnel for the implementation of clinical trials for the three most devastating infectious diseases in the region—HIV/AIDS, malaria, and tuberculosis. Fourteen participating European Union member states, plus Norway and Switzerland, are part of the governing assembly that decides the scientific priorities and overall strategy of the program. The strategic plan is developed by an independent panel working closely with a network of European scientific national programs and their African counterparts. This process ensures the input and commitment of the African countries and researchers and creates networks of European scientists who had previously been working independently on similar projects or in the same country.

The basis of EDCTP is a partnership model that helps the EU members integrate and coordinate their national research and development programs and encourages them to create links with African counterparts. North–South and South–South networking is promoted through mentorship with matched partners as a way to retain capacity and to ensure synergy and optimal use of resources. Besides funding trials on drugs and interventions to tackle the diseases on their priority list, recent EDCTP funding has included fellowships for African nationals, initiatives to build capacity for local ethics and regulatory committees, creation of networks of excellence for medical research, and establishment of clinical trial sites that include funding for local students and postdoctoral fellows. This integrated approach, which combines support for research and clinical trials with networking and capacity development, will most likely have a sustained impact in the region and will hopefully lead the way for other funding agencies working in the developing world.

Other organizations that continue to place emphasis on scientific capacity building include the WHO Programme for Research and Training in Tropical Diseases (TDR) [10],[11] and the Wellcome Trust (http://www.wellcome.ac.uk). Both organizations fund South-led research capacity networks and training workshops that focus on a sustainable approach to research in developing countries. Recently funded initiatives include the South–South joint programs in genomics training in Africa (AFROVECTEN) [12], the TDR-South African Bioinformatics Institute (SANBI) regional training center (http://www.ssi-tdr.net), and the Health Research Capacity Strengthening (HRCS) initiative in Kenya and Malawi.

To make a real and meaningful difference, the current interest and commitment to improving global health must be maintained over the long term. Importantly, the voices of our developing-country colleagues need to be heard and to direct the agenda. Building local capacity rather than focusing only on quantitative output is critical to making a lasting contribution. Because the human element—people-to-people connections—underlies the most successful partnerships, scaling up can also be envisioned as “scaling out,” where innumerable personal connections solidify and fortify the bridges between institutions and countries. Global health goals will be achievable when the necessary tools and knowledge are in the hands of our colleagues in the South, along with adequate human and physical infrastructure for health care and health research, supported by a dynamic international community.

## Abbreviations

EDCTP: European and Developing Countries Clinical Trials Partnership
HIT: health information technology
ICT: information and communication technologies
SSI: Sustainable Sciences Institute
